# Downregulation of oxytocin-related genes in periodontitis

**DOI:** 10.3389/fnmol.2022.950919

**Published:** 2022-08-24

**Authors:** Soudeh Ghafouri-Fard, Leila Gholami, Naghme Nazer, Bashdar Mahmud Hussen, Arezou Sayad, Mohammadreza Hajiesmaeili

**Affiliations:** ^1^Department of Medical Genetics, Shahid Beheshti University of Medical Sciences, Tehran, Iran; ^2^Department of Periodontics, Dental Research Center, Hamadan University of Medical Sciences, Hamadan, Iran; ^3^Department of Electrical Engineering, Sharif University of Technology, Tehran, Iran; ^4^Department of Pharmacognosy, College of Pharmacy, Hawler Medical University, Erbil, Iraq; ^5^Center of Research and Strategic Studies, Lebanese French University, Erbil, Iraq; ^6^Dental Research Center, Research Institute for Dental Sciences, Dental School, Shahid Beheshti University of Medical Sciences, Tehran, Iran; ^7^Critical Care Quality Improvement Research Center, Loghman Hakin Hospital, Shahid Beheshti University of Medical Sciences, Tehran, Iran

**Keywords:** periodontitis, oxytocin, *FOS*, *ITPR*, *RCAN1*, *RGS2*

## Abstract

Periodontitis is a common oral disorder leading to tooth loss in both developed and developing regions of the world. This multifactorial condition is related to the abnormal activity of several molecular pathways, among them are oxytocin-related pathways. In this study, we enrolled 26 patients and 28 controls and assessed the expression of four oxytocin-related genes, namely, *FOS*, *ITPR*, *RCAN1*, and *RGS2*, in circulation and affected tissues of enrolled individuals using real-time PCR. Expression of *FOS* was downregulated in total periodontitis tissues compared with total control tissues [ratio of mean expression (RME) = 0.23, *P*-value = 0.03]. Expression of *FOS* was also lower in total blood samples of patients compared with total controls. Expression of *ITPR* was downregulated in total periodontitis tissues compared with total control tissues (RME = 0.16, *P*-value = 0.01). Moreover, the expression of *ITPR* was reduced in total blood samples of patients compared with controls (RME = 0.25, *P*-value = 0.03). Expression of *RCAN1* was downregulated in total periodontitis tissues compared with total control tissues (RME = 0.17, *P*-value = 0.01). However, the expression of *RCAN1* was not different in blood samples of affected vs. unaffected individuals. Finally, the expression of *RGS2* was lower in total periodontitis tissues compared with total control tissues (RME = 0.24, *P*-value = 0.01) and in total blood samples of affected individuals compared with controls (RME = 0.42, *P*-value = 0.05). This study provides data about the association between expressions of oxytocin-related genes and the presence of periodontitis. Future studies are needed to unravel the mechanistic links and find the correlation between expressions of these genes and the pathological stage of periodontitis.

## Introduction

Periodontal diseases are common conditions in both developed and developing regions of the world with a global prevalence of 20–50% (Nazir, 2017). The chronic inflammatory disease of the periodontium is called periodontitis. This condition might result in the loss of periodontal ligament and damage to alveolar bone adjacent to the periodontium (de Pablo et al., 2009). As the principal cause of tooth loss, periodontitis is one of the major dangers to the health of the oral cavity (Benjamin, 2010). This condition is a multifactorial disorder with different etiologies related to host immune response, tissue destruction pathways, and bone resorption pathways (Qasim et al., 2020). Notably, transcriptomics analyses have shown a remarkable difference in the expression profiles of numerous genes between periodontitis and normal samples (Zhang et al., 2020). Oxytocin-related pathways have recently been found to be correlated with the pathogenesis of periodontitis. First, oxytocin has been shown to facilitate proliferation and enhance migratory potential and differentiation of periodontal stem cells into osteoblasts (Ge et al., 2019). Moreover, periodontal disorders have been considered possible risk factors for adverse pregnancy outcomes (Bansal et al., 2011). Prostaglandins, which are produced during the course of periodontitis (Båge et al., 2011), have also been shown to increase the number of oxytocin receptors in the myometrium (Sánchez, 2008). In addition, periodontal diseases have been found to increase systemic inflammatory responses and expressions of prostaglandin E2 and proinflammatory cytokines in pregnant women (Latorre Uriza et al., 2018). Based on these observations, we have hypothesized that the expression of oxytocin-related genes might be different between patients with periodontitis and normal subjects. To test this hypothesis, we designed this study and measured the expression levels of *FOS*, *ITPR*, *RCAN1*, and *RGS2* genes in the affected tissues and the circulation of patients with periodontitis vs. appropriate controls. These genes have been recently found to be correlated with oxytocin signaling through an *in silico* approach and have also been shown to be dysregulated in related disorders (Behtaji et al., 2021).

## Materials and methods

### Tissues and blood samples

Tissue samples were excised during surgical procedures from patients with chronic periodontitis (stages II–IV) according to the criteria previously described (Sayad et al., 2020a). Other criteria were age ≥18 years and the presence of a minimum of 16 teeth. Cases with a history of smoking, systemic disorders, intake of antibiotics or anti-inflammatory medicines, pregnancy, and breastfeeding were excluded. The presence of other inflammatory conditions was another exclusion criterion since we wanted to exclude the effects of inflammation in other sites on the expression of the mentioned genes. All cases were assessed by a university-affiliated periodontist. Control samples were obtained from bleeding on probing-free sites of individuals undergoing crown lengthening after careful examination by the periodontist. The study protocol was approved by the ethical committee of Shahid Beheshti University of Medical Sciences.

### Expression assays

Total RNA was isolated from tissues and blood specimens using PicoPure RNA Isolation Kit (Thermo Fisher Scientific). All steps were performed using instructions provided in the kit manual. Then, the cDNA synthesis kit (Smobio, Taiwan) was used to produce cDNA from RNA. Relative expressions (RE) of *FOS*, *ITPR*, *RCAN1*, and *RGS2* were quantified in all specimens using the qRT-PCR kit (GeneDireX, Miaoli County, Taiwan). Reactions were conducted on the LightCycler 96 instrument in duplicate. The *B2M* gene was used as a normalizer. Table 1 displays the sequences of primers used for the quantification of *FOS*, *ITPR*, *RCAN1*, and *RGS2* levels.

**TABLE 1:** Primer sequences.

Gene name	Sequence
*B2M*	F: AGATGAGTATGCCTGCCGTG R: GCGGCATCTTCAAACCTCCA
*FOS*	F: TACTACCACTCACCCGCAGA R: CGTGGGAATGAAGTTGGCAC
*ITPR*	F: GACGCAGTGCTACTCAACAAAC R: CAAATGCAGGAGCTGGATCAC
*RCAN1*	F: AGACTGAGTTTCTGGGAAAGGA R: CAGAAACTGCTTGTCTGGATTTG
*RGS2*	F: GGGAGAACGATAATGCAAAGTG R: AAGTAGCTCAAACGGGTCTTC

### Statistical methods

The R software and ggplot2, ggfortify, and ggpubr packages were used for data analyses. Transcript amounts of *FOS*, *ITPR*, *RCAN1*, and *RGS2* were measured from Ct and efficiency parameters. Gene expression data were normalized to transcript the levels of *B2M*. As gene expression figures were extremely skewed on a linear scale, a logarithmic transformation was performed to obtain parametric and accurate data. The significance of the difference in mean values of expressions of genes was evaluated using the *t*-test. The correlations between expression levels of *FOS*, *ITPR*, *RCAN1*, and *RGS2* were valued using the Spearman correlation test. Receiver operating characteristic (ROC) curves were spotted to measure the diagnostic values of expression levels of genes using pROC and the caret package. Youden’s J statistic was used to discover the optimum threshold. Area under curve (AUC) values were quantified.

## Results

This study was conducted on samples obtained from 26 patients with periodontitis and 28 controls. Table 2 shows the demographic data of cases and controls.

**TABLE 2:** General features of study participants.

Parameters	Periodontitis patients	Controls
Total number of tissue samples	26	28
Female	16	12
Male	10	16
Mean age ± SD	37.6 ± 2.5	37.5 ± 1.7
Total number of blood samples	23	17
Female	15	10
Male	8	7
Mean age ± SD	38.1 ± 2.9	37.9 ± 2.6

### Expression assays

**FIGURE 1:**
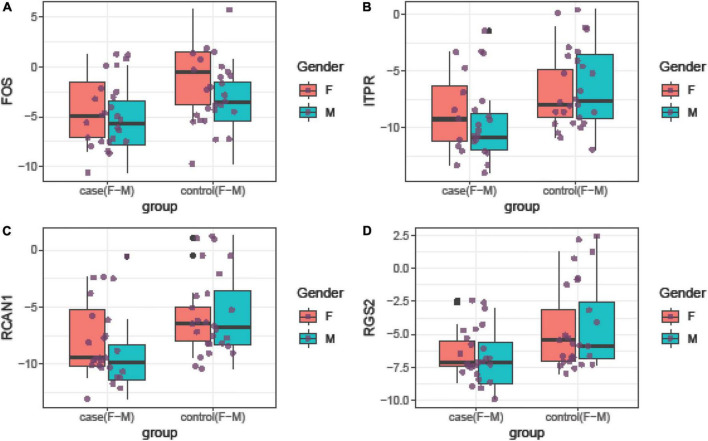
Relative expression amounts of *FOS* **(A)**, *ITPR* **(B)**, *RCAN1* **(C)**, and *RGS2* **(D)** genes in affected tissues compared with control tissues.

**FIGURE 2:**
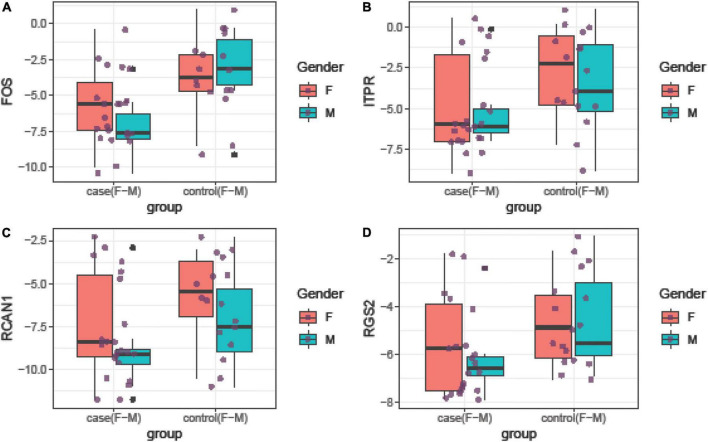
Relative expression amounts of *FOS* **(A)**, *ITPR* **(B)**, *RCAN1* **(C)**, and *RGS2* **(D)** genes in blood samples of patients with periodontitis compared with controls.

Relative expressions of *FOS*, *ITPR*, *RCAN1*, and *RGS2* genes in tissue and blood samples of patients and controls are depicted in Figures 1, 2, respectively.

Expression of *FOS* was downregulated in total periodontitis tissues compared with total control tissues (RME = 0.23, *P*-value = 0.03) and in affected tissues obtained from women compared with female control tissue (RME = 0.12, *P*-value = 0.03). Yet, this difference was not seen among men (*P*-value = 0.13). Expression of *FOS* was also lower in the total blood samples of patients compared with total controls (RME = 0.15, *P*-value < 0.001) and in blood samples of affected men compared with control men (RME = 0.07, *P*-value = 0.02). Among women, there was a trend toward downregulation of *FOS* in the blood samples of the affected individuals (RME = 0.23, *P*-value = 0.06). Expression of *ITPR* was down-regulated in total periodontitis tissues compared with total control tissues (RME = 0.16, *P*-value = 0.01) and in the affected tissues obtained from males compared with male control tissue (RME = 0.08, *P*-value = 0.02). Yet, this difference was not seen among females (*P*-value = 0.18). Moreover, the expression of *ITPR* was reduced in total blood samples of patients compared with controls (RME = 0.25, *P*-value = 0.03). Expression of *RCAN1* was downregulated in total periodontitis tissues compared with total control tissues (RME = 0.17, *P*-value = 0.01) and in affected tissues obtained from men compared with male control tissue (RME = 0.09, *P*-value = 0.03). However, the expression of *RCAN1* was not different in blood samples of affected vs. unaffected individuals. Finally, the expression of *RGS2* was lower in total periodontitis tissues compared with total control tissues (RME = 0.24, *P*-value = 0.01), affected tissues obtained from men compared with male control tissue (RME = 0.16, *P*-value = 0.02), and total blood samples of affected individuals compared with controls (RME = 0.42, *P*-value = 0.05). Table 3 shows the detailed results of the assessment of expression of *FOS*, *ITPR*, *RCAN1*, and *RGS2* genes in the tissue and blood specimens of periodontitis patients compared with controls.

**TABLE 3:** Statistical parameters of assessment of expression of *FOS*, *ITPR*, *RCAN1*, and *RGS2* genes in the tissues and blood specimens obtained from patients compared with controls.

		*FOS*					*ITPR*					*RCAN1*					*RGS2*				
Number of samples		SE	Ratio of mean expressions	*P*-Value	95% CI		SE	Ratio of mean expressions	*P*-Value	95% CI		SE	Ratio of mean expressions	*P*-Value	95% CI		SE	Ratio of mean expressions	*P*-Value	95% CI	
**Case/Control (Tissues)**																					
Total	26/28	0.92	0.23	**0.03**	−3.95	−0.25	0.96	0.16	**0.01**	−4.59	−0.73	0.96	0.17	**0.01**	−4.45	−0.61	0.74	0.24	**0.01**	−3.54	−0.54
F	16/12	1.36	0.12	**0.03**	−5.88	−0.24	1.27	0.30	0.18	−4.38	0.86	1.31	0.26	0.16	−4.64	0.80	1.05	0.36	0.18	−3.72	0.76
M	10/16	1.25	0.25	0.13	−4.61	0.65	1.47	0.08	**0.02**	−6.76	−0.63	1.48	0.09	**0.03**	−6.52	−0.32	1.11	0.16	**0.02**	−4.98	−0.38
**Case/Control (Blood)**																					
Total	23/17	0.84	0.15	**0.00**	−4.41	−0.99	0.89	0.25	**0.03**	−3.81	−0.18	0.88	0.32	0.07	−3.45	0.12	0.62	0.42	**0.05**	−2.52	0.00
F	15/10	1.06	0.23	0.06	−4.34	0.11	1.13	0.23	0.07	−4.48	0.20	1.07	0.30	0.12	−3.98	0.48	0.80	0.50	0.22	−2.68	0.65
M	8/7	1.38	0.07	**0.02**	−6.83	−0.73	1.49	0.28	0.24	−5.16	1.46	1.46	0.30	0.25	−4.93	1.43	1.01	0.31	0.12	−3.93	0.52

Bold values indicate significant p-values (<0.05).

**FIGURE 3:**
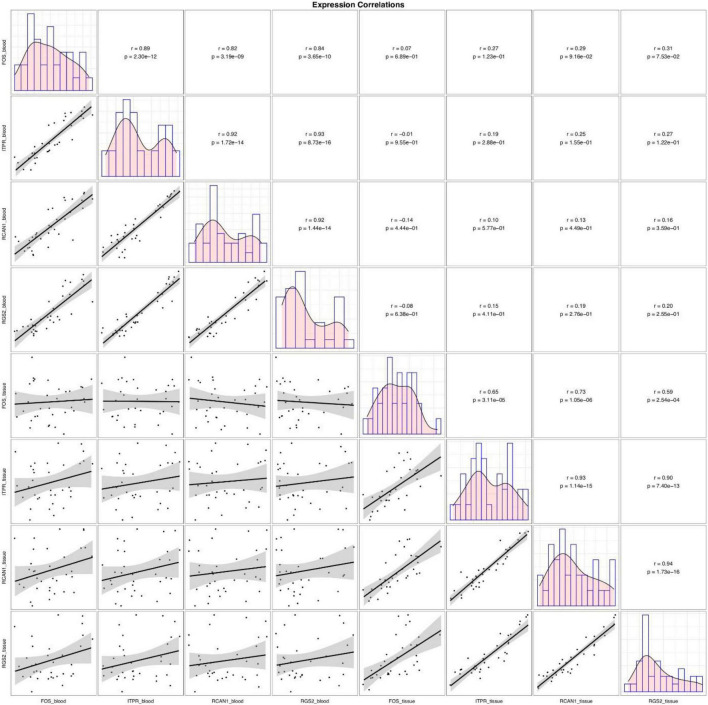
Correlations between tissue/blood levels of *FOS*, *ITPR*, *RCAN1*, and *RGS2* genes. The distributions of parameters are indicated on the diagonals. The bivariate scatter plots with fitted lines are shown in the inferior parts. Correlation coefficients and *P*-values are shown in the upper part of the diagonal.

Although expressions of *FOS*, *ITPR*, *RCAN1*, and *RGS2* genes were consistently correlated in each set of clinical samples (blood or tissue samples), their expressions in blood samples were not correlated with their expression in tissue samples (Figure 3).

**FIGURE 4:**
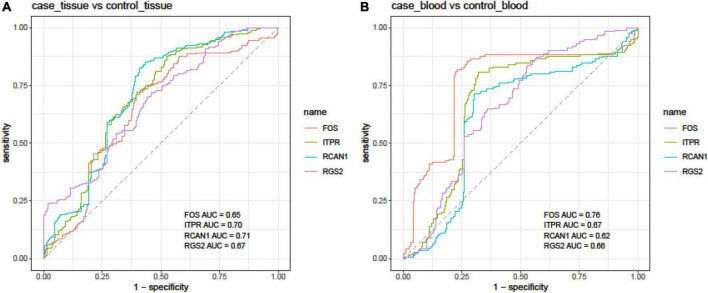
Receiver operating characteristic (ROC) curves depicted using the Bayesian generalized linear model.

Finally, we assessed the diagnostic power of *FOS*, *ITPR*, *RCAN1*, and *RGS2* genes in different sets of blood and tissue samples using the Bayesian generalized linear model (Figure 4).

In tissue samples, the best diagnostic power was achieved using the expression levels of *RCAN1* (AUC = 0.71, sensitivity = 0.81, and specificity = 0.59), followed by *ITPR* (AUC = 0.70, sensitivity = 0.87, and specificity = 0.47). In blood samples, *FOS* had the best performance in the separation of affected individuals from controls (AUC = 0.76, sensitivity = 0.82, and specificity = 0.77). The combination of expression levels of *FOS*, *ITPR*, *RCAN1*, and *RGS2* genes did not improve the diagnostic power either in tissue or blood samples (Table 4).

**TABLE 4:** Statistical parameters of ROC curves in tissue and blood samples.

		*FOS*			*ITPR*			*RCAN1*			*RGS2*			All		
Number of samples		AUC	Sensitivity	Specificity	AUC	Sensitivity	Specificity	AUC	Sensitivity	Specificity	AUC	Sensitivity	Specificity	AUC	Sensitivity	Specificity
**Case/Control (Tissue)**																
Total	26/28	0.65	0.71	0.60	0.70	0.87	0.47	0.71	0.83	0.59	0.67	0.72	0.54	0.65	0.82	0.44
**Case/Control (Blood)**																
Total	23/17	0.76	0.82	0.77	0.67	0.81	0.68	0.62	0.71	0.70	0.66	0.84	0.47	0.72	0.72	0.70

## Discussion

Periodontitis is a complex disorder associated with the dysregulation of several genes (Sayad et al., 2020a,b). Most of the assessed genes and pathways have been those related to the regulation of immune responses (Sayad et al., 2020a). However, it is clear that dysregulation of these pathways does not completely explain the pathogenic events during the course of this disorder (Sayad et al., 2020a). Thus, the identification of other relevant pathways in periodontitis is expected to improve our understanding of this disorder. Oxytocin has been shown to suppress inflammatory responses, induce antibiotic-like impacts, enhance wound healing and regenerative cascades, and inhibit stress-related immune diseases (Li et al., 2017). Moreover, several lines of evidence indicate an association between oxytocin-related pathways and periodontitis (Båge et al., 2011; Bansal et al., 2011; Ge et al., 2019). We have recently reported an association between a number of genes and oxytocin in the context of breast cancer (Behtaji et al., 2021). In this study, we assessed the expression of *FOS*, *ITPR*, *RCAN1*, and *RGS2* genes in the circulation and affected tissues of patients with periodontitis compared with normal controls.

Expression of *FOS* was downregulated in total periodontitis tissues compared with total control tissues and in affected tissues obtained from women compared with female control tissue. Yet, this difference was not seen among males. Expression of *FOS* was also lower in total blood samples of patients compared with total controls and in blood samples of affected men compared with male controls. Among women, there was a trend toward the downregulation of *FOS* in blood samples of affected individuals. FOS is a nuclear phosphoprotein that interacts with the JUN/AP-1 transcription factor. The complex formed between SMAD3, SMAD4, JUN, and FOS has a critical role in the regulation of TGF-β signaling (Zhang et al., 1998). Moreover, FOS participates in the regulation of the development of cells predestined to make and preserve the structure of the skeleton as well as immune system (Wagner and Eferl, 2005). Downregulation of this nuclear phosphoprotein in tissues and blood samples of patients with periodontitis might facilitate the development of this disorder through dysregulation of immune responses and interruption of the regenerative processes in the bone structures.

Expression of *ITPR* was downregulated in total periodontitis tissues compared with total control tissues and in affected tissues obtained from men compared with male control tissues. Yet, this difference was not observed among women. Moreover, the expression of *ITPR* was reduced in total blood samples of patients compared with controls. *ITPR1* has been shown to regulate autophagy and the sensitivity of cancer cells to chemotherapeutic drugs (Li et al., 2019). Notably, disruption in the regulatory mechanisms of autophagy has been demonstrated in periodontitis (Jiang et al., 2020). Meanwhile, numerous pharmaceutical and nutraceutical agents have been shown to modulate autophagy, thus serving as useful therapies for periodontitis (Lorenzo-Pouso et al., 2019).

Expression of *RCAN1* was downregulated in total periodontitis tissues compared with total control tissues and in affected tissues obtained from men compared with male control tissue. However, the expression of *RCAN1* was not different in blood samples of affected vs. unaffected individuals. RCAN1 has been shown to interact with calcineurin A and suppress calcineurin-related signaling pathways (Torac et al., 2014). Consistent with our results, expression of RCAN1 has been shown to be decreased in endothelial cells obtained from periodontal tissues affected by chronic inflammation (Peters et al., 2016).

Finally, the expression of *RGS2* was lower in total periodontitis tissues compared with total control tissues, affected tissues obtained from men compared with male control tissue, and total blood samples of affected individuals compared with controls. This gene has been shown to control signaling using G-protein coupled receptors (Nunn et al., 2010). This family of proteins has an important role in the development of bone diseases (Luo et al., 2019). Future studies are needed to appraise whether the contribution of RGS2 in the pathogenesis of periodontitis is exerted through the modulation of bone structure.

Moreover, we assessed the ability of these transcripts to separate affected tissues/blood samples from unaffected ones. In tissue samples, the best diagnostic power was achieved using the expression levels of *RCAN1* followed by the *ITPR*. In blood samples, *FOS* had the best performance in the separation of affected individuals from controls.

## Conclusion

This study provides data about the association between the expression of oxytocin-related genes and the presence of periodontitis and warrants upcoming studies to unravel the mechanistic links. Furthermore, we suggest an assessment of the correlation between the expression of these genes and the pathological stage of periodontitis.

## Data availability statement

The raw data supporting the conclusions of this article will be made available by the authors, without undue reservation.

## Ethics statement

The studies involving human participants were reviewed and approved by the study protocol was approved by the Ethics Committee of Shahid Beheshti University of Medical Sciences (IR.SBMU.DRC.REC.1400.018). The patients/participants provided their written informed consent to participate in this study.

## Author contributions

SG-F wrote the manuscript and revised it. MH and AS designed and supervised the study. BH and LG collected the data and performed the experiment. NN analyzed the data. All authors read and approved the submitted manuscript.
